# Identification of endophytic *Trichoderma* species (Hypocreaceae, Hypocreales) and their application against leaf blight of host *Platycladus orientalis*

**DOI:** 10.3897/mycokeys.128.174850

**Published:** 2026-02-19

**Authors:** Ning Jiao, Zhe Zhang, Kevin D. Hyde, Alvin M. C. Tang, Jinglan Liu, Jing Wang, Ablat Tohtirjap, Ying Zhang

**Affiliations:** 1 School of Ecology and Nature Conservation, Beijing Forestry University, Beijing 100083, China Center of Excellence in Fungal Research, Mae Fah Luang University Chaing Rai Thailand; 2 Center of Excellence in Fungal Research, Mae Fah Luang University, Chaing Rai 57100, Thailand School of Life Sciences, The Chinese University of Hong Kong Hong Kong China; 3 School of Life Sciences, The Chinese University of Hong Kong, Hong Kong SAR, China College of Life Sciences, Shihezi University Shihezi China; 4 Beijing Working People’s Cultural Palace, Beijing 100006, China School of Ecology and Nature Conservation, Beijing Forestry University Beijing China; 5 College of life Sciences, Shihezi University, Shihezi 832003, China Beijing Working People's Cultural Palace Beijing China

**Keywords:** *Alternaria* leaf blight, biocontrol, novel species, *Platycladus orientalis*, taxo­nomy, *
Trichoderma
*

## Abstract

Ancient *Platycladus orientalis* holds significant historical, cultural, ecological, and landscape value. Leaf blight caused by *Alternaria* spp. is the most common disease of *P. orientalis*. However, no reports are currently available on the screening and application of biocontrol agents for controlling this disease. In this study, several *Trichoderma* isolates were obtained from ancient *P. orientalis* in Beijing. Based on morphological characteristics and phylogenetic analyses of the concatenated ITS, *tef*1-α and *rpb*2 loci, four *Trichoderma* species were identified, including *T. citrinoviride*, *T. obovatum*, *T. nordicum*, and *T. platycladi*. Of these, *T. platycladi* is a species new to science, whereas *T. nordicum* and *T. obovatum* are new host records. Plate antagonism tests showed that representative strains of the four *Trichoderma* species exhibited significant inhibitory effects against colonies of *Alternaria alternata* and *A. cantlous*, the causal agents of leaf blight of ancient *P. orientalis*. Evaluation of the control effects on 1-year-old *P. orientalis* seedlings showed that these *Trichoderma* strains significantly controlled leaf blight, and *T. nordicum* CGMCC3.28772 exhibited the highest relative control effect under the same inoculation conditions.

## Introduction

Ancient trees are valuable natural resources and historical relics that sustain biodiversity and ecosystem functions, as well as provide social and cultural benefits to people (Huang et al. 2023). *Platycladus orientalis* (Cupressaceae), native to China and North Korea, is a robust, long-lived, evergreen coniferous tree widely distributed in China (Li et al. 2016; Dong et al. 2023). The ancient *P. orientalis*, symbolizing auspiciousness and longevity, is commonly found in prominent historical and cultural sites in Beijing, including palaces, temples, and parks. Beijing hosts the largest number of ancient trees in China, with 22,570 ancient *P. orientalis*, accounting for 55.69% of the city’s total ancient tree population (Zhao 2022; Zhang 2024).

Ancient *P. orientalis*, remaining in an over-mature stage for extended periods, experience declining physiological function and stress resistance, as well as urban human disturbances, which significantly increase the risk of diseases (Lindenmayer and Laurance 2017; Jiao et al. 2024). Leaf blight caused by *Alternaria* spp. is the most common disease of *P. orientalis*. As early as 1922, *P. orientalis* leaf blight caused by *A. pruni* was identified in northern China (Zhu 1922). Leaf blight of ancient *P. orientalis* caused by *A. tenuis* was reported in Beijing in 1996, resulting in extensive leaf and branch death (Lei et al. 1996). Subsequently, *A. alternata* was identified as the causal agent of leaf blight of ancient *P. orientalis* at the Mausoleum of the Yellow Emperor in Shaanxi (Li et al. 2021). In addition, *A. alternata* and *A. infectoria* were reported to cause leaf blight and dieback of *P. orientalis* around Tbilisi (Danelia et al. 2021). We have recently confirmed that leaf blight affecting ancient *P. orientalis* in Beijing is caused by *Alternaria* species, including *A. alternata*, *A. subcucurbitae*, *A. cantlous*, and *A. platycladi* (Jiao et al. 2025). Of these, *A. alternata* and *A. cantlous* were the most dominant causal agents (Jiao et al. 2025).

*Trichoderma* (Hypocreaceae, Hypocreales), originally introduced by Persoon in 1794, is an ecologically and economically important genus (Persoon 1794; Ye et al. 2023). Species of *Trichoderma* are widely distributed in various ecosystems, including natural soils, decaying wood, plant leaves, bark, and root systems, and also live as endophytes in plant tissues (Samuels 2006; Zheng et al. 2021). To date, more than 500 species have been described and recognized (Zhao et al. 2025). Many *Trichoderma* species are extensively used in agriculture, industry, and medicine, serving as bio-fungicides to control plant diseases, regulators of plant growth, fortifiers of soil fertility, and producers of antibiotics and enzymes (Saravanakumar and Kathiresan 2014; Bischof et al. 2016; Ye et al. 2023). Additionally, some members show great potential for applications in soil and water pollution remediation, as well as in the production of gold or silver nanoparticles (Harman et al. 2004; Anand et al. 2006; Mazyar et al. 2010). Endophytic *Trichoderma* species exhibit enhanced adaptation and colonization in their native hosts, thereby providing more effective disease control as biocontrol agents (Joseph 2025). For example, the endophytic *T. koningiopsis* T2, isolated from *Liriodendron chinense* × *tulipifera*, can effectively suppress black spot disease caused by *Alternaria alternata* and *Colletotrichum gloeosporioides* (Kong et al. 2022). In addition, a wettable powder formulation developed from the endophytic *T. zelobreve* T20 effectively controlled apple canker caused by *Cytospora cincta* and *Neoscytalidium dimidiatum* (Ranjbar et al. 2024). However, screening of endophytic *Trichoderma* species from *P. orientalis* for controlling leaf blight has not yet been studied.

In this study, several *Trichoderma* isolates were obtained from ancient *P. orientalis* in Beijing. The aim of this study was to (1) identify the *Trichoderma* isolates based on morphological characteristics and multi-locus phylogenetic analysis using ITS, *tef*1-α, and *rpb*2 loci, (2) determine the resistance of these representative *Trichoderma* strains against *Alternaria* pathogens through plate antagonism tests, and (3) evaluate the control effect of these *Trichoderma* strains against *Alternaria* leaf blight of *P. orientalis* through in situ inoculation in a greenhouse.

## Materials and methods

### Specimen collection and fungal isolation

Healthy branch and trunk tissues of ancient *P. orientalis* were collected from three locations (Zhongshan Park, Fahai Temple, and Working People’s Cultural Palace) in Beijing, China, from September 2023 to December 2024. The tissue was cut into 0.5 × 0.5 × 0.2 cm sections, then surface-sterilized with 75% ethanol for 60 s and 1% NaOCl for 60 s, followed by three rinses with sterile distilled water, and cultured on potato dextrose agar (PDA) medium at 25 °C until fungal colonies were observed (Zhou et al. 2019). Pure cultures were obtained from hyphal tips at the margins of single colonies, which were subcultured on fresh PDA and maintained at 25 °C. Based on colony characteristics, several *Trichoderma* isolates were obtained. The specimens were deposited at Beijing Forestry University (BJFU), whereas the fungal isolates were submitted to the China General Microbiological Culture Collection Center (CGMCC).

### Morphological characterization

The purified strains were cultured on PDA (10 g potato extract, 20 g dextrose, 13 g agar, 1 L distilled water), malt extract agar (MEA; 20 g malt extract, 15 g agar, 1 L distilled water), cornmeal dextrose agar (CMD; 40 g cornmeal, 20 g dextrose, 15 g agar, 1 L distilled water), and synthetic low-nutrient agar (SNA; 1 g KH_2_PO_4_, 1 g KNO_3_, 0.5 g MgSO_4_, 0.5 g KCl, 0.2 g glucose, 0.2 g sucrose, 15 g agar, 1 L distilled water) for 7 days in an incubator at 25 °C with alternating 12 h/12 h fluorescent light/darkness (Zhao et al. 2023). Colony diameters were measured after 3 days, and trials were replicated three times. The morphological characteristics of colonies, including colony appearance, color, radial and concentric rings formed by spore production, pigmentation, and odor, were recorded (Zheng et al. 2021). The characteristics of conidiophores, conidia, and phialides were photographed, and at least 30 measurements per structure were documented and examined under a microscope (Nikon Eclipse E600).

### DNA extraction, PCR amplification, and sequencing

Mycelia (approximately 0.1 g) were subjected to DNA extraction using a CTAB plant genomic DNA fast extraction kit (Aidlab Biotechnologies Co., Ltd., Beijing, China). The internal transcribed spacer (ITS) region was amplified with primers ITS5 and ITS4 (White et al. 1990), *tef*1-α with primers EF1-728F (Carbone and Kohn 1999) and EF1-LLErev (Jaklitsch et al. 2005), and *rpb*2 with primers fRPB2-5f and fRPB2-7cr (Liu et al. 1999). The polymerase chain reaction (PCR) amplification system and reaction conditions were described by Zhao et al. (2025). PCR amplicons were purified and sequenced at Sangon Biotechnology Co., Ltd. (Beijing, China).

### Phylogenetic analyses

The newly generated sequences in this study were submitted to GenBank to obtain accession numbers, and ITS, *tef*1-α, and *rpb*2 sequences of closely related *Trichoderma* species were retrieved from GenBank for phylogenetic analysis (Suppl. material 1). Multiple sequence alignment was performed using MAFFT v.7.110 (http://mafft.cbrc.jp/alignment/server/). Ambiguous sequences at the start and end were deleted and manually adjusted using BioEdit.

A combined dataset of ITS, *tef*1-α, and *rpb*2 sequences from *Trichoderma* species was used to perform phylogenetic analyses based on maximum likelihood (ML), Bayesian inference (BI), and maximum parsimony (MP). ML analyses were conducted using RAxML-HPC BlackBox v.8.2.10 under the GTR+GAMMA model with 1,000 bootstrap replicates (Stamatakis 2014). BI analyses were performed in MrBayes v.3.2.6 with four Markov Chain Monte Carlo (MCMC) chains run for 5,000,000 generations, sampling every 1,000 generations and discarding the initial 25% of sampled data as burn-in (Ronquist et al. 2012). MP analyses were performed in PAUP* v.4.0b10 using heuristic searches with tree bisection–reconnection (TBR) branch swapping and 1,000 random addition replicates (Swofford 2002). Phylogenetic trees were visualized using FigTree v.1.4.4 (http://tree.bio.ed.ac.uk/software/figtree).

### Plate antagonism tests

According to our recent report (Jiao et al. 2025), representative pathogenic strains of leaf blight on ancient *P. orientalis* in Beijing, *Alternaria alternata* CGMCC3.28778 and *A. cantlous* CGMCC3.28780, were selected for further study. The tested biocontrol strains were representative strains of four *Trichoderma* species isolated in this study: *T. citrinoviride* CGMCC3.28773, *T. nordicum* CGMCC3.28772, *T. obovatum* CGMCC3.28771, and *T. platycladi* CGMCC3.28769. All tested strains were deposited at BJFU.

The dual-culture plate method was used for antagonism tests (Ojaghian 2011). Mycelial plugs with an 8 mm diameter of *Trichoderma* and *Alternaria* strains were cut from the edges of 5-day-old colonies. The plugs were placed at both ends of PDA plates (50 mm apart) and incubated at 25 °C. Plates inoculated only with the *Alternaria* strain served as the control. The colony diameter of the pathogen was measured daily, and the inhibition rate was calculated after 5 days. The inhibition rate was calculated as follows (Zhang et al. 2018): mycelial growth inhibition rate (%) = (control pathogen colony diameter − treatment pathogen colony diameter) / (control pathogen colony diameter − mycelial plug diameter) × 100%.

All experiments were carried out three times in parallel and repeated three times.

### Evaluation of control efficacy

Leaves were inoculated via spray inoculation with a 10^8^ spores/mL *Trichoderma* sp. suspension and smear inoculation with a 10^6^ spores/mL *Alternaria* sp. suspension. Each *Trichoderma* strain treatment corresponded one-to-one with each *Alternaria* pathogen inoculation. The pot experiments were divided into four groups as follows (Kong et al. 2022): treatment 1 involved inoculating *Trichoderma* one day before the pathogen; treatment 2 involved simultaneous inoculation of *Trichoderma* and the pathogen; treatment 3 involved inoculating *Trichoderma* one day after the pathogen; and the control consisted of *Alternaria* pathogen inoculation only. One-year-old *P. orientalis* seedlings (aboveground height, approximately 25 cm) were selected for inoculation experiments. Six leaf clusters per pot were inoculated, and the selected leaf clusters in each pot had similar areas and numbers of scale leaves (approximately 30 scale leaves per pot). Leaf surfaces were slightly wounded before inoculation. One day after inoculation with *Alternaria* spore suspensions, slight water-soaked symptoms were observed on the leaves. Infection of the leaves was confirmed by fulfilling Koch’s postulates. The inoculated *P. orientalis* seedlings were placed in a greenhouse at 14 °C–28 °C, 50%–75% relative humidity, under a 12 h light/dark cycle, and disease development was monitored daily. Each treatment had three repetitions with three pots each.

In the greenhouse experiment to investigate the biological control effect of *Trichoderma* spp. on *P. orientalis* leaf blight, incidence and disease index were quantified after 7 days of treatment. Disease was quantified according to diseased leaf area (Table 1) as follows (Kong et al. 2022): incidence rate (%) = number of diseased leaves / total number of leaves × 100%; disease index = (Σ representative value at all levels × leaf number at all levels) / (highest representative value × total leaf number) × 100; relative control effect (%) = (control disease index − disease index of each treatment) / control disease index × 100%.

**Table 1.** Grading standard for leaf blight of *Platycladus orientalis*.

Disease grade	Grading standard	Representative value
0	Asymptomatic	0
I	The area of disease spots occupies 1 to 5% of the foliar area	1
II	The area of disease spots occupies 5 to 10% of the foliar area	2
III	The area of disease spots occupies 10 to 20% of the foliar area	3
IV	The area of disease spots occupies 20 to 50% of the foliar area	4
V	Leaf disease spots area > 50%	5

### Statistical analysis

Data were analyzed using an analysis of variance and Duncan’s multiple comparison tests with IBM SPSS Statistics 19 (IBM Inc., Armonk, NY). Standard errors of all mean values were calculated (*P* < 0.05).

## Results

### Phylogenetic analysis

In the *tef*1-α phylogenetic tree, strains CGMCC3.28769 and CGMCC3.28770 formed a distinct clade within the Harzianum clade and appeared closely related to *T. inhamatum* (Suppl. material 2). The strain CGMCC3.28773 clustered with *T. citrinoviride* in the Longibrachiatum clade. Strains CGMCC3.28771 and CGMCC3.28772 clustered with *T. obovatum* and *T. nordicum*, respectively, in the Atroviride clade. In the *rpb*2 phylogenetic tree, strains CGMCC3.28769 and CGMCC3.28770 formed a distinct clade within the Harzianum clade, clustering close to *T. afarasin*, *T. azevedoi*, *T. camerunense*, *T. lentiforme*, and *T. shaanxiensis* (Suppl. material 3). The positions of strains CGMCC3.28773 and CGMCC3.28771 were consistent with those in the *tef*1-α phylogenetic tree, whereas CGMCC3.28772 clustered with *T. nigricans* and *T. nordicum* in the Atroviride clade.

**Figure 1.**
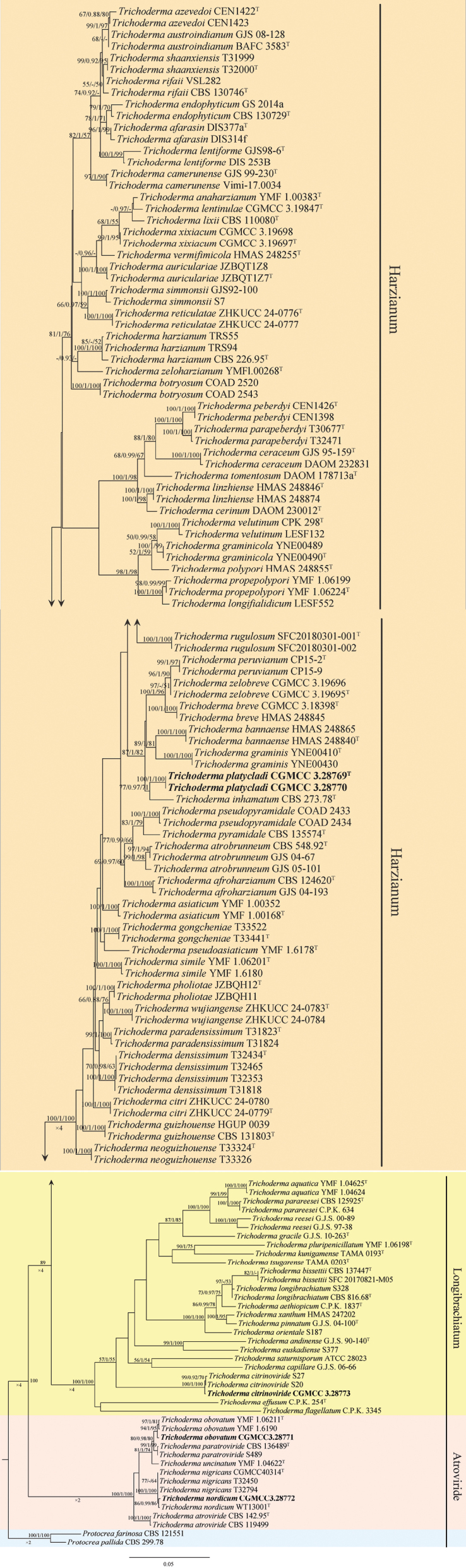
Phylogenetic tree generated by maximum likelihood analysis using the concatenated sequences of the ITS, *tef*1-α, and *rpb*2 loci of the genus *Trichoderma*. RAxML bootstrap support values (ML ≥ 50%), Bayesian posterior probability (PP ≥ 0.90), and MP bootstrap support values (ML ≥ 50%) are shown at the nodes. Strains in this study are highlighted in bold.

The phylogenetic tree based on the concatenated ITS, *tef*1-α, and *rpb*2 datasets, analyzed using three methods (ML, BI, and MP), revealed the classification of one new species in the Harzianum clade, one known species in the Longibrachiatum clade, and two known species in the Atroviride clade (Fig. 1). In the phylogram, *T. platycladi* formed a distinct clade within the Harzianum clade with strong support (ML/BI/MP = 100/1/100). The strain CGMCC3.28773 within the Longibrachiatum clade clustered with *T. citrinoviride* with high support (ML/BI/MP = 100/1/100). In the Atroviride clade, strain CGMCC3.28772 formed a well-supported clade with *T. nordicum* (ML/BI/MP = 86/0.99/86), and strain CGMCC3.28771 clustered with *T. obovatum* with high support (ML/BI/MP = 94/1/95).

### Taxonomy

#### Trichoderma citrinoviride

Taxon classificationFungiHypocrealesHypocreaceae

Bissett, Canad. J. Bot. 62(5): 926 (1984)

12084BC6-BCDA-5606-AB8C-CEE862C92CBD

Fig. 2: Part I(i)

##### Strains examined.

China • Beijing, Shijingshan District, Fahai Temple (39°56'29"N, 116°9'33"E), on the branch of ancient *P. orientalis*, December 2024, N. Jiao and Z. Zhang (BJFU-FHS354; living culture: CGMCC3.28773).

##### Notes.

Phylogenetic analysis showed that strain CGMCC3.28773 clustered together with *T. citrinoviride* in the Longibrachiatum clade, with strong support (ML/BI/MP = 100/1/100). Morphologically, the characteristics and sizes of phialides (3.6–6.8 × 2.4–3.0 μm, n = 30 in this study vs. 3.5–6.6 × 2.0–3.2 μm), conidia (2.9–3.7 × 1.8–2.4 μm, n = 50 in this study vs. 2.2–3.7 × 1.5–2.1 μm), and chlamydospores (5.8–7.0 × 3.5–5.0 μm, n = 30 in this study vs. 4–7 × 3–5 μm) were similar to the description provided by Bissett (1984) (Table 2). Based on phylogeny and morphology, this isolate was identified as *T. citrinoviride*. *Trichoderma citrinoviride* has previously been reported as an endophyte of *P. orientalis*, and in this study, this species was isolated again.

**Figure 2.**
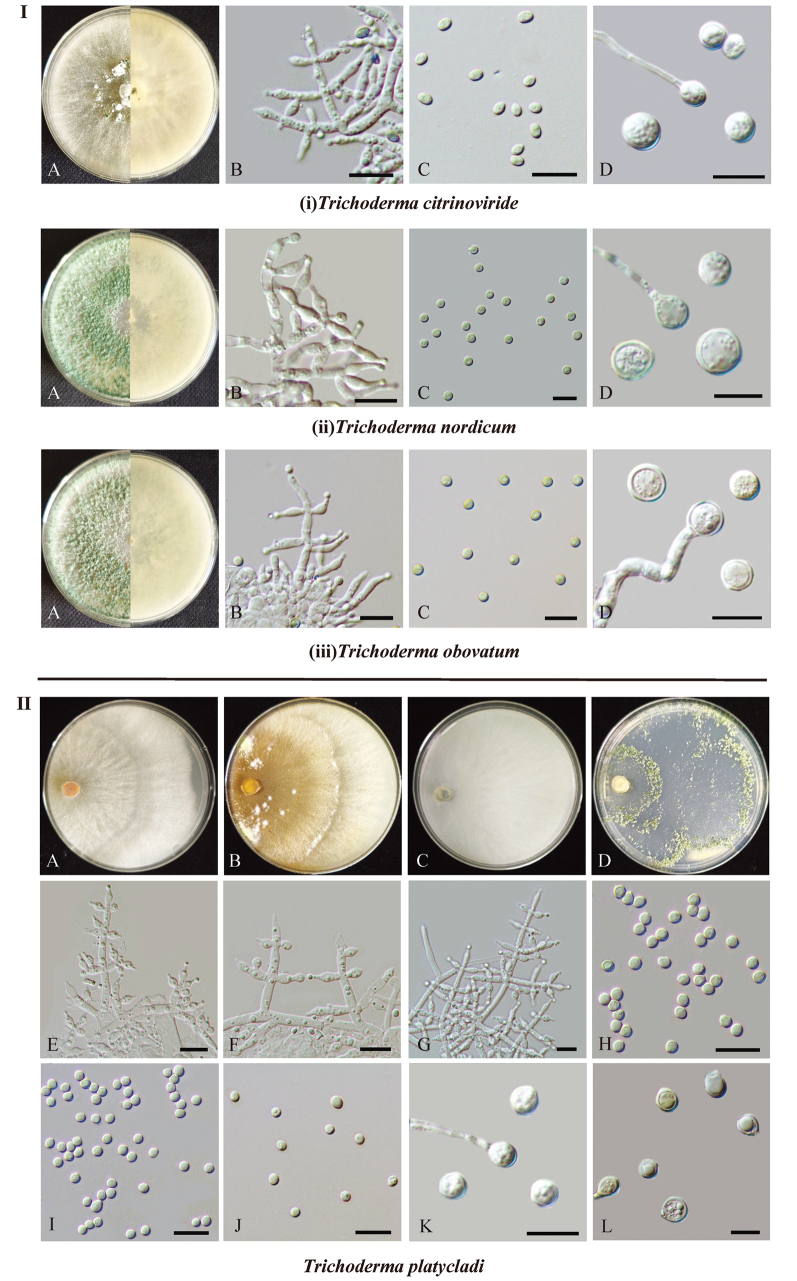
Colonies and microscopic characteristics of *Trichoderma* species isolated from the ancient *P. orientalis*. **Part I**: the morphology of the three known species. **A**. Colonies after 7 d at 25 °C on PDA; **B**. Conidiophores and phialides; **C**. Conidia; **D**. Chlamydospore. Scale bars: 10 μm (**B–D**). **Part II**: the morphology of the novel species *T. platycladi*. **A–D**. Colonies after 7 d at 25 °C on media (A: PDA; B: MEA; C: CMD; D: SNA); **E–G**. Conidiophores and phialides; **H–J**. Conidia; **K, L**. Chlamydospore. Scale bars: 10 μm (**E–L**).

**Table 2.** Morphological characteristics of *Trichoderma* species in this study.^x^

Species	Strain	Colony character on PDA		Conidiophores	Phialides		Conidia		Chlamydospores	
		Description of colonial morphology	Diameters (mm/3 days)	Character	Size (μm)	Character	Size (μm)	Character	Size (μm)	Character
*T. citrinoviride*	CGMCC3.28773	White at first, quickly shading to green, occasionally forming compact, hemispherical pustules up to 10 mm in diameter, odor indistinct.	59–62	Irregularly shaped tufts, main branches long and relatively straight, side branches arising at near right angles, usually arising singly and at irregular intervals, or occasionally in opposite pairs, rarely branched.	3.6–6.8 × 2.4–3.0 (n = 30)	lageniform to ampulliform	2.9–3.7 × 1.8–2.4 (n = 50)	dilute green, more or less ellipsoidal	5.8–7.0 × 3.5–5.0 (n = 30)	single or in pairs, globose to subglobose
*T. nordicum*	CGMCC3.28772	Grey-white at first and slowly turning green, diffusing pigment or distinctive odour absent.	67–71	Branches narrow and flexuous, typically forming a pyramidal structure with regularly whorled branches, each branch terminating in a cruciate whorl of up to 5 phialides.	7.5–11.2 × 2.8–3.3 (n = 30)	lageniform	4.0–4.8 × 4.0–4.3 (n = 50)	green, globose to obovoidal	8.5–10.0 × 8.5–9.7 (n = 30)	sometimes present, globose to subglobose
*T. obovatum*	CGMCC3.28771	Pale green to green, margin dense, sparse in the middle, floccose, homoge neous, indistinctly zonate, aerial hyphae abundant, especially pleasant odor noted.	80–85	Straight or curved, emerging in right angles or oriented towards to the conidiophore axis, typically with 1–2 branched levels, side branches simple.	5.0–9.0 × 2.8–4.0 (n = 30)	ampuliform to lageniform, sometimes nearly round	3.2–4.0 × 3.0–3.5 (n = 50)	green, globose, oval to obovate	7.0–8.8 × 6.8–8.2 (n = 30)	single, terminal or intercalary, globose to subglobose
*T. platycladi*	CGMCC3.28769, CGMCC3.28770	Radial, white, fluffy, aerial hyphae dense, abundant, yellow-green and white conidial pustules form after 7 days, no distinct odor or diffusing pigment observed.	62–65	Pyramidal, with opposite branches slightly inclined upward or at right angles, sometimes arising at irregular intervals along the main axis, closely spaced and not re-branching.	6.0–8.1 × 3.0–3.8 (n = 30)	lageniform to subulate	2.6–3.4 × 2.4–2.8 (n = 50)	green, globose to obovoidal	5.1–7.0 × 4.9–6.8 (n = 30)	often terminal, globose to subglobose

^x^ Colony diameters on PDA under 25 °C in the dark were measured on the 3^rd^ day.

#### Trichoderma nordicum

Taxon classificationFungiHypocrealesHypocreaceae

G.Z. Zhang, MycoKeys 87: 144 (2022)

5891F2F5-C1B8-5343-A50A-1F0C7FB3C841

Fig. 2: Part I(ii)

##### Strains examined.

China • Beijing, Shijingshan District, Fahai Temple (39°56'29"N, 116°9'33"E), on the trunk of ancient *P. orientalis*, November 2024, N. Jiao and Z. Zhang (BJFU-FHS298; living culture: CGMCC3.28772).

##### Notes.

*Trichoderma nordicum* was originally isolated from soil in Hebei, China. Phylogenetic analysis revealed that strain CGMCC3.28772 clustered with the type strain *T. nordicum* WT13001 in the Atroviride clade. Morphologically, the type strain is characterized by lageniform phialides (7.2–10.3 × 2.9–3.2 μm) and globose to obovoidal conidia (4.4–4.8 × 4.1–4.4 μm), which are similar to those of our strain (Zhang et al. 2022) (Table 2). Based on both phylogenetic and morphological evidence, this strain was identified as *T. nordicum*. This study reports the first isolation of *T. nordicum* from *P. orientalis*.

#### Trichoderma obovatum

Taxon classificationFungiHypocrealesHypocreaceae

Z.F. Yu & Y.F. Lv, J. Fungi 7(6):467 (2021)

E3388C88-B9FC-5B7B-A469-069D6D1F3608

Fig. 2: Part I(iii)

##### Strains examined.

China • Beijing, Dongcheng District, Working People’s Cultural Palace (39°54'45"N, 116°23'54"E), on the trunk of ancient *P. orientalis*, November 2023, N. Jiao and Z. Zhang (BJFU-TM298; living culture: CGMCC3.28771).

##### Notes.

*Trichoderma obovatum* was originally isolated from soil in Yunnan, China. Phylogenetically, strain CGMCC3.28771 clustered with *T. obovatum* in the Atroviride clade with strong support values of 94/1/95 (ML/BI/MP). Morphologically, our isolate was consistent with the type strain, with phialides (5.0–9.0 × 2.8–4.0 μm, n = 30 in this study vs. 4.8–8.9 × 2.5–3.9 μm) and conidia (3.2–4.0 × 3.0–3.5 μm, n = 50 in this study vs. 3.2–3.8 × 3.0–3.6 μm) showing similar characteristics and sizes (Zheng et al. 2021) (Table 2). Therefore, this strain was identified as *T. obovatum*. To our knowledge, this is the first report of this species isolated from *P. orientalis*.

#### Trichoderma platycladi

Taxon classificationFungiHypocrealesHypocreaceae

N. Jiao & Y. Zhang sp. nov.

73F77AF7-0B96-590E-8E5D-07D9A97992F3

MycoBank No: 859234

Fig. 2: Part II

##### Etymology.

The epithet refers to the host plant genus *Platycladus*.

##### Description.

***Sexual morph***: Unknown. ***Asexual morph***: Conidiophores pyramidal, with opposite branches slightly inclined upward or at right angles, sometimes arising at irregular intervals along the main axis, closely spaced, and not re-branching. Phialides lageniform to subulate, often with a narrow neck, discrete or integrated, solitary or in whorls of 2–4, 5.8–8.1(−8.5) × 2.8–4.0 μm (mean ± SD = 7.5 ± 0.7 × 3.2 ± 0.2 μm, n = 30), length/width ratio of 1.8–2.9 (mean 2.3 ± 0.3), base 1.4–1.8 μm (mean ± SD = 1.5 ± 0.2 μm, n = 30). Conidia globose to obovoidal, green, 2.4–3.5 × 2.2–2.9 μm (mean ± SD = 3.1 ± 0.2 × 2.7 ± 0.1 μm, n = 50), length/width ratio of 1.0–1.3 (mean ± SD = 1.1 ± 0.1). Chlamydospores often terminal, globose to subglobose, 5.0–7.1 × 4.8–7.0 μm (mean ± SD = 6.0 ± 0.5 × 5.5 ± 0.6 μm, n = 30), length/width ratio of 1.0–1.2 (mean ± SD = 1.1 ± 0.1).

##### Culture characteristics.

Optimal growth at 30 °C. Colony radius on PDA after 3 days: 62–65 mm at 25 °C, covering the plate at 30 °C, and 30–32 mm at 35 °C. Covering the plate after 4 days at 25 °C. Colony radial, white, fluffy, aerial hyphaedense, abundant. Yellow-green and white conidial pustules form after 7 days. No distinct odor or diffusing pigment observed. Colony radius on MEA after 3 days: 56–58 mm at 25 °C, 68–70 mm at 30 °C, and 28–31 mm at 35 °C. Covering the plate after 4 days at 25 °C and at 30 °C. Colony well-defined, radial, mycelia white, forming a broad distinct zonate after 7 days. No distinct odor or diffusing pigment observed. Colony radius on CMD after 3 days: 40–45 mm at 25 °C, 62–65 mm at 30 °C, and 36–38 mm at 35 °C. Covering the plate after 5 days at 25 °C and 4 days at 30 °C. Colony white, radial, aerial hyphae rare. No distinct odor or diffusing pigment observed. Colony radius on SNA after 3 days: 40–43 mm at 25 °C, 62–65 mm at 30 °C, and 25–27 mm at 35 °C. Covering the plate after 5 days at 25 °C and 4 days at 30 °C. Colony hyaline, radial, aerial hyphae sparse. Pale green and white conidial pustules form after 3 days, a small green disk around the inoculums. No distinct odor or diffusing pigment observed.

##### Type.

China • Beijing, Dongcheng District, Zhongshan Park (39°54'37"N, 116°23'39"E), on the trunk of ancient *P. orientalis*, September 2023, N. Jiao and Y. Zhang (holotype BJFU-ZS17; paratype BJFU-ZS15, ex-type culture: CGMCC3.28769).

##### Additional strain examined.

• Living culture: CGMCC3.28770; other information is the same as the type.

##### Notes.

*Trichoderma platycladi* is recognized as an endophyte of *P. orientalis*. Phylogenetic analysis indicated that this species is a new member of the Harzianum clade and is closely related to *T. inhamatum*. *T. platycladi* exhibits nucleotide differences from *T. inhamatum* in the *rpb*2 region amounting to 3.2% (25/788, one gap) and differences of 1.1% (6/563, two gaps) in *tef*1-α. Morphologically, the phialides of *T. platycladi* are obviously longer than those of the type strain of *T. inhamatum* (mean 7.5 × 3.2 μm vs. 5.0 × 3.2 μm) (Veerkamp and Gams 1983; Chaverri et al. 2015). In addition, the conidia of *T. platycladi* are slightly larger than those of *T. inhamatum* (2.6–3.4 × 2.4–2.8 μm vs. 2.5–3.0 × 2.2–2.7 μm) (Veerkamp and Gams 1983; Chaverri et al. 2015). Based on morphological characteristics and multigene phylogenetic analyses, this species is described as a novel taxon.

### Plate antagonism test

**Figure 3.**
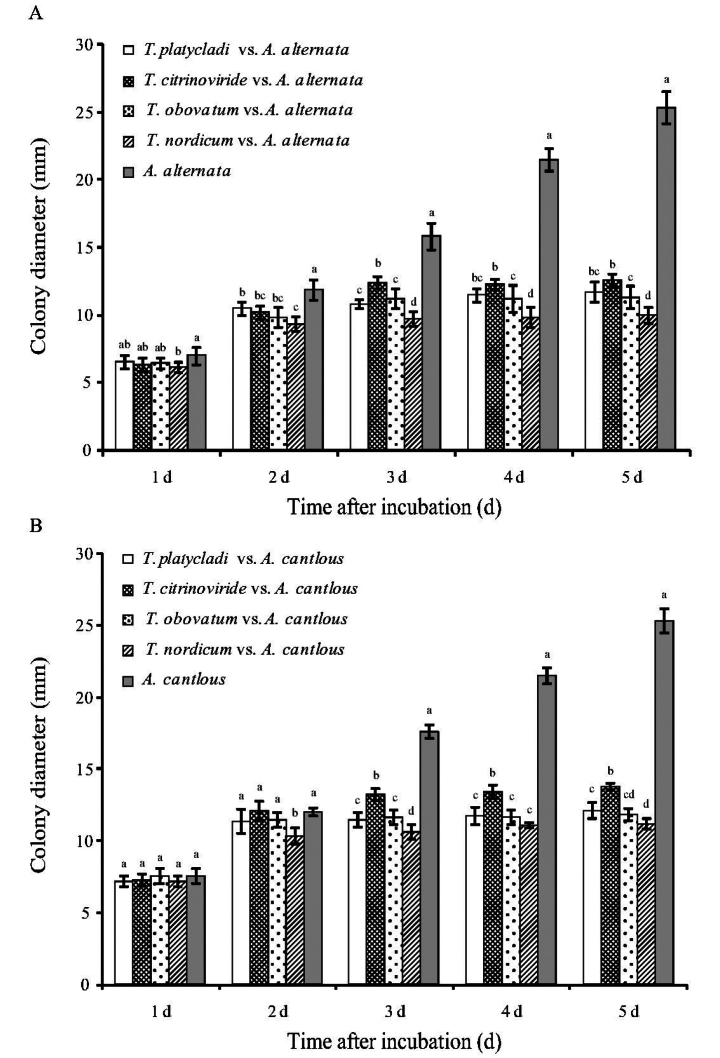
Plate antagonism test of the four representative *Trichoderma* strains against *Alternaria* pathogens. **A**. Colony diameters of *A. alternata* CGMCC3.28778 from 1 d to 5 d; **B**. Colony diameters of *A. cantlous* CGMCC3.28780 from 1 d to 5 d. Different letters indicate significant differences between the control and inoculation treatments based on Duncan’s post hoc test (*P* < 0.05).

From the second day after inoculation, four *Trichoderma* strains showed significant inhibitory effects against *A. alternata* (*P* < 0.05). By the third day, these strains also showed significant inhibition against *A. cantlous* (*P* < 0.05). The diameter of the pathogens in the *Trichoderma* strain treatments tended to stabilize from the third day, whereas the diameter of the pathogens in the control continued to increase (Fig. 3). On the fifth day, the inhibition rates of *T. platycladi* CGMCC3.28769, *T. citrinoviride* CGMCC3.28773, *T. obovatum* CGMCC3.28771, and *T. nordicum* CGMCC3.28772 against *A. alternata* were 52.30%, 45.39%, 53.29%, and 55.92%, respectively, whereas the inhibition rates against *A. cantlous* were 53.95%, 50.33%, 55.26%, and 60.53%, respectively. Based on the 5-day antagonism tests, *T. nordicum* CGMCC3.28772 showed the strongest antagonistic effect against *Alternaria* pathogens, followed by *T. platycladi* CGMCC3.28769 and *T. obovatum* CGMCC3.28771, whereas *T. citrinoviride* CGMCC3.28773 showed the weakest inhibition.

### Evaluation of control efficacy

*In situ* inoculation on 1-year-old *P. orientalis* seedlings in the greenhouse demonstrated that the relative control effect against *Alternaria* leaf blight exceeded 75% when the *Trichoderma* strain was inoculated before the pathogen and still exceeded 50% when the *Trichoderma* strain was inoculated after the pathogen (Table 3). The disease index when the *Trichoderma* strain was inoculated before the pathogen was significantly lower than when the *Trichoderma* strain was inoculated after the pathogen (*P* < 0.05), whereas the relative control effect was significantly higher (*P* < 0.05). The evaluation results showed that application of *T. nordicum* CGMCC3.28772 resulted in the lowest disease index and the highest relative control effect under the same inoculation conditions compared with the other three *Trichoderma* strains, indicating that it is the most effective biocontrol strain.

**Table 3.** The evaluation of the control efficacy of *Trichoderma* spp. against *Alternaria* leaf blight of *Platycladus orientalis* in the greenhouse. ^y^

Inoculated strains	Inoculation condition	Incidence rate (%)		Disease index		Relative control effect (%)	
		*A. alternata* CGMCC3.28778	*A. cantlous* CGMCC3.28780	*A. alternata* CGMCC3.28778	*A. cantlous* CGMCC3.28780	*A. alternata* CGMCC3.28778	*A. cantlous* CGMCC3.28780
*T. platycladi* CGMCC3.28769	Before pathogens	10.05 ± 0.63 H	10.34 ± 1.46 g	14.67 ± 2.07 FG	12.67 ± 4.68 g	83.38 ± 2.69 AB	85.32 ± 6.60 a
	Simultaneously	15.57 ± 0.59 DE	15.94 ± 1.35 ef	21.33 ± 4.13 E	22.00 ± 4.20 ef	76.63 ± 3.13 CD	75.11 ± 5.26 bc
	After pathogens	32.15 ± 1.74 C	34.10 ± 2.62 bc	34.67 ± 4.84 BC	36.67 ± 3.01 bc	60.64 ± 6.40 FG	58.50 ± 6.07 ef
*T. citrinoviride* CGMCC3.28773	Before pathogens	14.03 ± 0.84 EF	10.54 ± 1.89 g	19.33 ± 3.01 EF	15.33 ± 3.01 fg	78.15 ± 3.24 BCD	82.54 ± 4.32 ab
	Simultaneously	13.22 ± 0.71 FG	12.42 ± 1.28 fg	28.67 ± 3.01 D	20.67 ± 5.89 f	67.37 ± 5.20 E	76.37 ± 8.12 b
	After pathogens	30.14 ± 0.90 C	30.24 ± 0.92 cd	37.33 ± 4.13 B	34.67 ± 3.27 cd	57.56 ± 6.52 G	60.74 ± 6.17 de
*T. obovatum* CGMCC3.28771	Before pathogens	11.45 ± 1.02 GH	10.31 ± 0.94 g	15.33 ± 3.01 FG	16.67 ± 3.93 fg	82.53 ± 4.03 ABC	81.33 ± 4.27 ab
	Simultaneously	16.57 ± 0.79 D	18.79 ± 0.57 e	22.67 ± 5.47 E	28.67 ± 3.01 de	74.50 ± 5.22 D	67.67 ± 4.53 cd
	After pathogens	39.54 ± 0.74 B	36.24 ± 3.77 b	38.67 ± 4.84 B	42.67 ± 4.13 b	56.42 ± 3.91 G	52.13 ± 3.10 f
*T. nordicum* CGMCC3.28772	Before pathogens	9.49 ± 1.33 H	10.49 ± 0.35 g	10.67 ± 3.26 G	11.33 ± 3.93 g	86.64 ± 4.54 A	86.84 ± 5.80 a
	Simultaneously	11.74 ± 1.77 FGH	12.24 ± 1.17 fg	18.67 ± 3.27 EF	17.33 ± 4.84 fg	78.96 ± 3.28 BCD	80.39 ± 5.81 ab
	After pathogens	31.58 ± 1.37 C	29.52 ± 0.51 d	30.67 ± 3.27 CD	32.00 ± 3.58 cd	65.33 ± 3.71 EF	63.56 ± 7.38 de
*Alternaria* pathogens	Alone	50.65 ± 1.68 A	48.29 ± 4.82 a	88.67 ± 6.89 A	89.33 ± 9.35 a	—	—
—	Sterile water	0 I	0 h	0 H	0 h	—	—

^y^ Different letters indicate significant differences, with *P* < 0.05 according to Duncan’s post hoc test.

## Discussion

Four endophytic *Trichoderma* species were identified from ancient *P. orientalis* in Beijing, namely *T. citrinoviride*, *T. nordicum*, *T. obovatum*, and *T. platycladi*. *Trichoderma citrinoviride* was previously reported as an endophyte from the leaves and branches of *P. orientalis* during a survey of conifers in Tbilisi and its surroundings (Danelia et al. 2021). Subsequently, endophytic *T. harzianum* was isolated from the branches of ancient *P. orientalis* in Shaanxi, China (Yu et al. 2021). In addition, Hosseyni-Moghaddam and Soltani (2014) reported that endophytic *T. atroviride* and *T. koningii* from Cupressaceae plants effectively inhibited common cypress fungal pathogens, including *Diplodia seriata*, *Phaeobotryon cupressi*, and *Spencermartinsia viticola*. Bu et al. (2016) isolated 311 endophytic fungal strains from the leaves of ancient *P. orientalis* in Shaanxi, which were classified into 21 genera, with *Trichoderma* as the dominant genus. Among these, *Trichoderma* sp. C-14 exhibited significant inhibitory effects against four plant pathogenic fungi: *Cytospora chrysosperma*, *Fusarium oxysporum*, *Colletotrichum gloeosporioides*, and *Botryosphaeria dothidea*. In the present study, *T. citrinoviride* was re-isolated as an endophyte from *P. orientalis*, and three other *Trichoderma* species were isolated, enriching the diversity of endophytic *Trichoderma* associated with *P. orientalis*.

The representative strains of the four *Trichoderma* species exhibited strong antagonistic activity against *Alternaria* pathogens in this study. Many members of *Trichoderma* can efficiently inhibit *Alternaria* pathogens. For example, *T. harzianum* significantly inhibited the growth of *A. alternata*, the causal agent of tobacco brown spot (Gveroska and Ziberoski 2012). Furthermore, the endophytic *T. citrinoviride* isolated from *Panax ginseng* exhibited strong antifungal activity against *Alternaria panax* (Park et al. 2019). Evaluation of the biocontrol effect demonstrated that the four *Trichoderma* strains were effective in controlling leaf blight of *P. orientalis*, with the highest effect observed when the *Trichoderma* strains were applied one day prior to inoculation with *Alternaria* pathogens. Comparable results have been reported for the control of tobacco black shank by *T. harzianum* CGMCC23294 and the control of leaf black spot in *L. chinense* × *tulipifera* by *T. koningiopsis* T2, in which the application of *Trichoderma* strains before pathogen inoculation achieved the best disease control (Ren et al. 2025; Kong et al. 2022). This may be due to the ability of *Trichoderma* spp. to colonize leaf surfaces rapidly and establish dominant populations in advance, thereby delaying disease occurrence (Guzmán-Guzmán et al. 2023). Moreover, among the tested strains, *T. nordicum* CGMCC3.28772 showed the highest effect, highlighting its potential for controlling leaf blight of *P. orientalis*.

## Supplementary Material

XML Treatment for Trichoderma citrinoviride

XML Treatment for Trichoderma nordicum

XML Treatment for Trichoderma obovatum

XML Treatment for Trichoderma platycladi

## Additional information

### Use of AI

No use of AI was reported.

### Author ORCIDs

Ning Jiao https://orcid.org/0009-0004-2858-9731

Zhe Zhang https://orcid.org/0009-0001-8425-7246

Kevin D. Hyde https://orcid.org/0000-0002-2191-0762

Alvin M. C. Tang https://orcid.org/0000-0001-5055-5221

Ablat Tohtirjap https://orcid.org/0009-0007-1944-7506

Ying Zhang https://orcid.org/0000-0001-8817-6032

### Data availability

All of the data that support the findings of this study are available in the main text or Supplementary Information. All sequences have been deposited in GenBank under the accession numbers provided in the text.

## Supplementary materials

Supplementary material 1 Isolates and GenBank accession numbers of *Trichoderma* species used for phylogenetic analysis Data type docx https://binary.pensoft.net/file/1543350This dataset is made available under the Open Database License (http://opendatacommons.org/licenses/odbl/1.0/). The Open Database License (ODbL) is a license agreement intended to allow users to freely share, modify, and use this Dataset while maintaining this same freedom for others, provided that the original source and author(s) are credited. Ning Jiao, Zhe Zhang, Kevin D. Hyde, Alvin M.C. Tang,Jinglan Liu, Jing Wang, Ablat Tohtirjap, Ying Zhang

Supplementary material 2 Phylogenetic tree generated by maximum likelihood analysis using the tef1-α gene sequences of the genus *Trichoderma* Data type png Explanation note RAxML bootstrap support values (ML ≥ 50%), Bayesian posterior probability (PP ≥ 0.90), and MP bootstrap support values (ML ≥ 50%) are shown at the nodes. Strains in this study are highlighted in bold. https://binary.pensoft.net/file/1543351This dataset is made available under the Open Database License (http://opendatacommons.org/licenses/odbl/1.0/). The Open Database License (ODbL) is a license agreement intended to allow users to freely share, modify, and use this Dataset while maintaining this same freedom for others, provided that the original source and author(s) are credited. Ning Jiao, Zhe Zhang, Kevin D. Hyde, Alvin M.C. Tang,Jinglan Liu, Jing Wang, Ablat Tohtirjap, Ying Zhang

Supplementary material 3 Phylogenetic tree generated by maximum likelihood analysis using the *rpb*2 gene sequences of the genus *Trichoderma* Data type png Explanation note RAxML bootstrap support values (ML ≥ 50%), Bayesian posterior probability (PP ≥ 0.90), and MP bootstrap support values (ML ≥ 50%) are shown at the nodes. Strains in this study are highlighted in bold. https://binary.pensoft.net/file/1543352This dataset is made available under the Open Database License (http://opendatacommons.org/licenses/odbl/1.0/). The Open Database License (ODbL) is a license agreement intended to allow users to freely share, modify, and use this Dataset while maintaining this same freedom for others, provided that the original source and author(s) are credited. Ning Jiao, Zhe Zhang, Kevin D. Hyde, Alvin M.C. Tang,Jinglan Liu, Jing Wang, Ablat Tohtirjap, Ying Zhang
