# Supplementary material for: Identification of endophytic Trichoderma species (Hypocreaceae, Hypocreales) and their application against leaf blight of host Platycladus orientalis
Source: MycoKeys. 2026 Feb 19;128:331–50. doi: 10.3897/mycokeys.128.174850 (PMC12946831; doi:10.3897/mycokeys.128.174850)
Supplement: Supplementary material 1 — Isolates and GenBank accession numbers of Trichoderma species used for phylogenetic analysis [file mycokeys-128-331-s001.docx]

**Supplementary material 1:** Isolates and GenBank accession numbers of *Trichoderma* species used for phylogenetic analysis.

| Species name | Strain number | GenBank accession numbers | | |
| --- | --- | --- | --- | --- |
|  |  | ITS | *tef1-α* | *rpb2* |
| *Trichoderma aethiopicum* | C.P.K. 1837^T^ | — | — | HM182986 |
| *Trichoderma afarasin* | DIS 377a^T^ | FJ442665 | FJ463322 | FJ442799 |
| *Trichoderma afarasin* | DIS314f | FJ442259 | FJ463400 | FJ442778 |
| *Trichoderma afroharzianum* | CBS 124620^T^ | FJ442265 | FJ463301 | FJ442691 |
| *Trichoderma afroharzianum* | GJS 04–193 | FJ442233 | FJ463298 | FJ442709 |
| *Trichoderma anaharzianum* | YMF 1.00383^T^ | NR174890 | MH183182 | MH158995 |
| *Trichoderma andinense* | G.J.S. 90-140^T^ | — | AY956321 | JN175531 |
| *Trichoderma aquatica* | YMF 1.04624 | MH383057 | MK775506 | MK775511 |
| *Trichoderma aquatica* | YMF 1.04625^T^ | MH383058 | MK775507 | MK775512 |
| *Trichoderma asiaticum* | YMF 1.00168^T^ | MH262582 | MH236492 | MH262575 |
| *Trichoderma asiaticum* | YMF 1.00352 | MH113930 | MH183183 | MH158994 |
| *Trichoderma atrobrunneum* | CBS 548.92^T^ | AF443924 | AF443942 | — |
| *Trichoderma atrobrunneum* | GJS 04–67 | FJ442273 | FJ463360 | FJ442724 |
| *Trichoderma atrobrunneum* | GJS 05–101 | FJ442677 | FJ463392 | FJ442745 |
| *Trichoderma atroviride* | CBS 119499 | FJ860726 | FJ860611 | FJ860518 |
| *Trichoderma atroviride* | CBS 142.95^T^ | AY380906 | AY376051 | EU341801 |
| *Trichoderma auriculariae* | JZBQT1Z7^T^ | — | ON649896 | ON649949 |
| *Trichoderma auriculariae* | JZBQT1Z8 | — | ON649897 | ON649950 |
| *Trichoderma austroindianum* | BAFC 3583^T^ | — | MH352421 | — |
| *Trichoderma austroindianum* | GJS 08- 128 | — | MH352423 | — |
| *Trichoderma azevedoi* | CEN1422^T^ | MK714902 | MK696660 | MK696821 |
| *Trichoderma azevedoi* | CEN1423 | MK714903 | MK696661 | MK696822 |
| *Trichoderma bannaense* | HMAS 248840^T^ | KY687923 | KY688037 | KY687979 |
| *Trichoderma bannaense* | HMAS 248865 | KY687948 | KY688038 | KY688003 |
| *Trichoderma bissettii* | SFC 20170821-M05 | — | MN307414 | — |
| *Trichoderma bissettii* | CBS 137447^T^ | KJ174235 | HG931266 | — |
| *Trichoderma botryosum* | COAD 2520 | — | MK044140 | MK044233 |
| *Trichoderma botryosum* | COAD 2543 | — | MK044141 | MK044234 |
| *Trichoderma breve* | CGMCC 3.18398^T^ | KY687927 | KY688045 | KY687983 |
| *Trichoderma breve* | HMAS 248845 | KY687928 | KY688046 | KY687984 |
| *Trichoderma camerunense* | GJS 99–230^T^ | AY027780 | AF348107 | — |
| *Trichoderma camerunense* | Vimi-17.0034 | — | MZ675908 | MZ675862 |
| *Trichoderma capillare* | GJS 06-66 | — | JN175585 | JN175530 |
| *Trichoderma ceraceum* | DAOM 232831 | — | KJ871239 | — |
| *Trichoderma ceraceum* | G.J.S. 95–159^T^ | AF275332 | AY937437 | AF545508 |
| *Trichoderma cerinum* | DAOM 230012^T^ | — | KJ871242 | KJ842184 |
| *Trichoderma citri* | ZHKUCC24-0779^T^ | PP789092 | PP848435 | PP848451 |
| *Trichoderma citri* | ZHKUCC24-0780 | PP789093 | PP848436 | PP848452 |
| *Trichoderma citrinoviride* | S20 | — | KJ665449 | KJ665250 |
| *Trichoderma citrinoviride* | S27 | — | KJ665450 | KJ665251 |
| ***Trichoderma citrinoviride*** | **CGMCC 3.28773** | **PV473743** | **PV478073** | **PV491718** |
| *Trichoderma densissimum* | T31818 | — | OP357967 | OP357965 |
| *Trichoderma densissimum* | T32465 | — | OP357972 | OP357963 |
| *Trichoderma densissimum* | T32353 | — | OP357970 | OP357964 |
| *Trichoderma densissimum* | T32434 = CGMCC 3.24126^T^ | — | OP357971 | OP357966 |
| *Trichoderma effusum* | C.P.K. 254^T^ | — | KJ665473 | KJ665260 |
| *Trichoderma endophyticum* | CBS 130729^T^ | FJ442243 | FJ442243 | — |
| *Trichoderma endophyticum* | GS 2014a | FJ884177 | FJ967822 | — |
| *Trichoderma euskadiense* | S377 | — | KJ665492 | KJ665269 |
| *Trichoderma flagellatum* | C.P .K.3345 | — | FJ763158 | JN258689 |
| *Trichoderma gongcheniae* | T33522 | — | OR779519 | OR779490 |
| *Trichoderma gongcheniae* | T33441 = GDMCC 3.1011^T^ | — | OR779518 | OR779489 |
| *Trichoderma gracile* | G.J.S. 10-263^T^ | — | JN175598 | JN175547 |
| *Trichoderma graminicola* | YNE00489 | — | OR779520 | OR779493 |
| *Trichoderma graminicola* | YNE00490 = GDMCC 3.1015^T^ | — | OR779521 | OR779494 |
| *Trichoderma graminis* | YNE00430 | — | OR779515 | OR779492 |
| *Trichoderma graminis* | YNE00410 = GDMCC 3.1013^T^ | — | OR779514 | OR779491 |
| *Trichoderma guizhouense* | HGUP 0039 | JX089584 | JX089585 | JQ901401 |
| *Trichoderma guizhouense* | CBS 131803^T^ | JN191311 | JN215484 | JQ901400 |
| *Trichoderma harzianum* | TRS55 | KP009211 | KP008803 | KP009121 |
| *Trichoderma harzianum* | TRS94 | KP009250 | KP008802 | KP009120 |
| *Trichoderma harzianum* | CBS 226.95^T^ | — | AF348101 | AF545549 |
| *Trichoderma inhamatum* | CBS 273.78^T^ | FJ442680 | AF348099 | FJ442725 |
| *Trichoderma kunigamense* | TAMA 0193^T^ | AB807633 | AB807645 | AB807657 |
| *Trichoderma lentiforme* | GJS98-6^T^ | AF469189 | AF469195 | — |
| *Trichoderma lentiforme* | DIS 253B | FJ44261 | FJ851875 | FJ442756 |
| *Trichoderma lentinulae* | CGMCC 3.19847^T^ | MN594469 | MN605878 | MN605867 |
| *Trichoderma linzhiense* | HMAS 248874 | KY687957 | KY688048 | KY688011 |
| *Trichoderma linzhiense* | HMAS 248846^T^ | KY687929 | KY688047 | KY687985 |
| *Trichoderma lixii* | CBS110080^T^ | NR131264 | AF443938 | KJ665290 |
| *Trichoderma longibrachiatum* | CBS 816.68^T^ | MH859229 | EU401591 | DQ087242 |
| *Trichoderma longibrachiatum* | S328 | JQ685875 | JQ685867 | JQ685883 |
| *Trichoderma longifialidicum* | LESF552 | KT278901 | KT279020 | KT278955 |
| *Trichoderma neoguizhouense* | T33326 | — | OR779517 | OR779488 |
| *Trichoderma neoguizhouense* | T33324 = GDMCC 3.1012^T^ | — | OR779516 | OR779487 |
| *Trichoderma nigricans* | T32450 | — | OP357973 | OP357958 |
| *Trichoderma nigricans* | T32794 | — | OP357975 | OP357960 |
| *Trichoderma nigricans* | T32781 = CGMCC40314^T^ | — | OP357974 | OP357959 |
| *Trichoderma nordicum* | WT13001 =ACCC 39713^T^ | MH287483 | MH287501 | MH287502 |
| ***Trichoderma nordicum*** | **CGMCC3.28772** | **PV473742** | **PV478072** | **PV491717** |
| *Trichoderma obovatum* | YMF 1.06211^T^ | MN977803 | MT070144 | MT038432 |
| *Trichoderma obovatum* | YMF 1.6190 | — | MT070143 | MT038433 |
| ***Trichoderma obovatum*** | **CGMCC3.28771** | **PV473741** | **PV478071** | **PV491716** |
| *Trichoderma orientale* | S187 | JQ685873 | JQ685868 | JQ685884 |
| *Trichoderma paradensissimum* | T31823 = CGMCC 3.24125^T^ | — | OP357968 | OP357962 |
| *Trichoderma paradensissimum* | T31824 | — | OP357969 | OP357961 |
| *Trichoderma parapeberdyi* | T32471 | — | OR779511 | OR779484 |
| *Trichoderma parapeberdyi* | T30677 = GDMCC 3.1016^T^ | — | OR779510 | OR779483 |
| *Trichoderma parareesei* | CBS 125925^T^ | MH863773 | GQ354353 | HM182963 |
| *Trichoderma parareesei* | C.P.K. 634=TUB F-430 | — | GQ354351 | HM182968 |
| *Trichoderma paratroviride* | CBS 136489^T^ | — | KJ665627 | KJ665321 |
| *Trichoderma paratroviride* | S489 | — | KJ665628 | KJ665322 |
| *Trichoderma peberdyi* | CEN1398 | MK714874 | MK696632 | MK696794 |
| *Trichoderma peberdyi* | CEN1426^T^ | MK714906 | MK696664 | MK696825 |
| *Trichoderma peruvianum* | CP15-2^T^ | — | MW480145 | MW480153 |
| *Trichoderma peruvianum* | CP15-9 | — | MW480146 | MW480154 |
| *Trichoderma pholiotae* | JZBQH11 | — | ON649918 | ON649971 |
| *Trichoderma pholiotae* | JZBQH12^T^ | ON653405 | ON649919 | ON649972 |
| *Trichoderma pinnatum* | G.J.S. 04-100^T^ | — | JN175571 | JN175515 |
| ***Trichoderma platycladi*** | **CGMCC 3.28769^T^** | **PV473739** | **PV478069** | **PV491714** |
| ***Trichoderma platycladi*** | **CGMCC 3.28770** | **PV473740** | **PV478070** | **PV491715** |
| *Trichoderma pluripenicillatum* | YMF 1.06198^T^ | MN977788 | MT070159 | MT070160 |
| *Trichoderma polypori* | HMAS 248855^T^ | KY687938 | KY688058 | KY687994 |
| *Trichoderma propepolypori* | YMF 1.06199 | — | MT070157 | MT052182 |
| *Trichoderma propepolypori* | YMF 1.06224^T^ | — | MT070158 | MT052181 |
| *Trichoderma pseudoasiaticum* | YMF 1.6178^T^ | — | MT070155 | MT052183 |
| *Trichoderma pseudopyramidale* | COAD2433 | — | MK044157 | MK044250 |
| *Trichoderma pseudopyramidale* | COAD2434 | — | MK044158 | MK044251 |
| *Trichoderma pyramidale* | CBS 135574^T^ | — | KJ665699 | KJ665334 |
| *Trichoderma reesei* | G.J.S. 00-89 | — | JN175599 | JN175548 |
| *Trichoderma reesei* | G.J.S. 97-38 | AJ004962 | JN175603 | JN175552 |
| *Trichoderma reticulatae* | ZHKUCC24-0776^T^ | PP789089 | PP848432 | PP848448 |
| *Trichoderma reticulatae* | ZHKUCC24-0777 | PP789090 | PP848433 | PP848449 |
| *Trichoderma rifaii* | VSL282 | — | MZ476195 | — |
| *Trichoderma rifaii* | CBS 130746 = DIS 355B^T^ | FJ442663 | FJ463324 | — |
| *Trichoderma rugulosum* | SFC20180301-001^T^ | MH050353 | MH025984 | MH025986 |
| *Trichoderma rugulosum* | SFC20180301-002 | — | MH025985 | MH025987 |
| *Trichoderma saturnisporum* | ATCC 28023 | X93977 | JN388897 | JN175524 |
| *Trichoderma shaanxiensis* | T32000 = GDMCC 3.1014^T^ | — | OR779513 | OR779486 |
| *Trichoderma shaanxiensis* | T31999 | — | OR779512 | OR779485 |
| *Trichoderma simile* | YMF1.6180 | — | MT070153 | MT052185 |
| *Trichoderma simile* | YMF 1.06201^T^ | MN977793 | MT070154 | MT052184 |
| *Trichoderma simmonsii* | S7 | — | KJ665719 | KJ665337 |
| *Trichoderma simmonsii* | GJS92-100 | AF443919 | AF443937 | FJ442710 |
| *Trichoderma tomentosum* | DAOM 178713a^T^ | EU330958 | EU279969 | AF545557 |
| *Trichoderma tsugarense* | TAMA 0203^T^ | AB807635 | AB807647 | AB807659 |
| *Trichoderma uncinatum* | YMF 1.04622^T^ | MK795994 | MK795986 | MK795990 |
| *Trichoderma velutinum* | LESF132 | KT278865 | KT279019 | KT278954 |
| *Trichoderma velutinum* | CPK 298 = DAOM 230013^T^ | — | KJ665769 | KF134794 |
| *Trichoderma vermifimicola* | HMAS 248255^T^ | NR171951 | MN605882 | MN605871 |
| *Trichoderma wujiangense* | ZHKUCC24-0783^T^ | PP789096 | PP848439 | PP848455 |
| *Trichoderma wujiangense* | ZHKUCC24-0784 | PP789097 | PP848440 | PP848456 |
| *Trichoderma xanthum* | HMAS 247202 | — | MF371226 | MF371211 |
| *Trichoderma xixiacum* | CGMCC3.19697^T^ | MN594476 | MN605885 | MN605874 |
| *Trichoderma xixiacum* | CGMCC3.19698 | MN594477 | MN605886 | MN605875 |
| *Trichoderma zelobreve* | CGMCC 3.19696 | — | MN605884 | MN605873 |
| *Trichoderma zelobreve* | CGMCC 3.19695^T^ | — | MN605883 | MN605872 |
| *Trichoderma zeloharzianum* | YMFl.00268^T^ | NR165872 | MH183181 | MH158996 |
| *Protocrea farinosa* | CBS 121551 | MH863119 | EU703889 | EU703935 |
| *Protocrea pallida* | CBS 299.78 | MH861137 | EU703900 | EU703948 |

Notes: T, type strains. — , not applicable.
